# Economy of Effort or Sophisticated Programming? The Prevalence of Bidirectional Promoter Complexes in the Human Genome

**DOI:** 10.3390/genes15020252

**Published:** 2024-02-18

**Authors:** Erik M. Anderson, Stephen K. Anderson

**Affiliations:** 1 Cancer Innovation Laboratory, Center for Cancer Research, National Cancer Institute, Frederick, MD 21702, USA; erik.anderson3@nih.gov; 2 Basic Science Program, Frederick National Laboratory for Cancer Research, Frederick, MD 21702, USA

**Keywords:** antisense transcription, bidirectional promoters, lineage-determining transcription factors, noncoding RNAs

## Abstract

An abundance of antisense promoters in the vicinity of the transcriptional start site of coding genes suggests that they play an important role in gene regulation. The divergent transcription of housekeeping genes by a common central promoter region allows for coordinated regulation of genes in related pathways and is also linked to higher promoter activity. However, closely positioned transcription start sites can also result in competition between overlapping promoter elements and generate a binary switch element. Furthermore, the direct competition resulting from the presence of an antisense promoter immediately downstream of the transcription start site of the gene produces an element that can exist in only one of two stable transcriptional states: sense or antisense. In this review, we summarize analyses of the prevalence of antisense transcription in higher eukaryotes and viruses, with a focus on the antisense promoters competing with the promoters of coding genes. The structures of bidirectional promoters driving the simultaneous expression of housekeeping genes are compared with examples of human bidirectional elements that have been shown to act as switches. Since many bidirectional elements contain a noncoding RNA as the divergent transcript, we describe examples of functional noncoding antisense transcripts that affect the epigenetic landscape and alter the expression of their host gene. Finally, we discuss opportunities for additional research on competing sense/antisense promoters, uncovering their potential role in programming cell differentiation.

## 1. The Prevalence of Antisense Transcription in Protein-Coding Genes Indicates a Role in Gene Regulation

Antisense transcription is present in a substantial proportion of human and mouse genes, with more than 25% containing antisense transcripts that overlap exons of the coding gene (cis-antisense pairs), ~20% that contain non-overlapping antisense (intronic antisense), and a further 10% of genes that contain antisense transcripts upstream of the gene promoter (bidirectionally promoted pairs) [1]. The percentage of genes containing overlapping sense and antisense transcripts likely exceeds 40%, based on studies of Expressed Sequence Tags (EST) and Rapid Amplification of cDNA Ends (RACE) data [1,2]. In a study of human cell lines and peripheral blood mononuclear cells, 8–16% of expressed genes contained antisense transcripts [3]. Overlapping sense/antisense transcripts can modulate gene expression by multiple mechanisms, such as inducing DNA methylation, histone modification, the generation of silencing RNA, and the masking of microRNA (miRNA) binding sites or RNA destabilizing signals in 3′ untranslated regions by the formation of double-stranded RNA [4].

**Figure 1:**
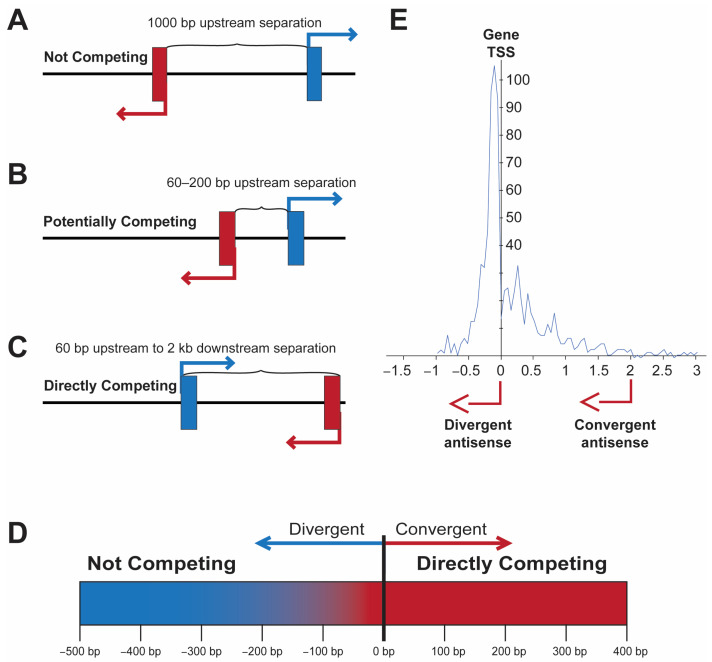
The location of an antisense TSS has distinct effects on gene transcription. (**A**–**C**) Depiction of three examples of antisense transcription near the TSS of a coding gene. (**A**) Non-competitive location. (**B**) Antisense upstream within 200 bp, potential competition of TF-binding sites. (**C**) Direct competition due to the overlap of RNA polymerase-binding region or head-to-head transcription. (**D**) Graphic illustrating the transition from non-competitive to competitive transcription ~60 bp upstream of the gene TSS. (**E**) Pattern of antisense TSS observed in human transcription factor genes. The distance in kb from the gene TSS is shown on the x-axis, and the number of genes with a TSS at a given position is shown on the y-axis. Adapted from Li et al. [7].

Antisense promoters located near the transcription start site (TSS) of coding genes may enhance the level or stochasticity of expression in the case of divergent transcription or potentially create binary switches if transcripts are convergent. Divergent promoters within 1 kb of each other are found in 11% of human genes, and 23% of these (2.5% of all genes) have overlapping 5′ ends, potentially creating bistable switches due to the direct competition of the RNA polymerase complexes [5]. A recent study by Rosikiewicz et al. [6] looked at 5′ overlap of transcripts from protein-coding genes in a head-to-head configuration. They did not find any evidence for competitive inhibition of the overlapping transcripts. However, they did not examine expression at the single-cell level. A more dynamic competition may occur in cases where divergent promoters have overlapping transcription factor (TF)-binding sites, or if there is insufficient space for two RNA polymerases to bind at the same time if the competing TSS are too close. Thus, the actual point at which the direct competition of transcription occurs is not when the antisense TSS is downstream of the sense TSS (Figure 1). The examination of the distance between divergent transcripts in human genes, as well as human transcription factors (Figure 1E), reveals that most bidirectional promoters have start sites separated by more than 60 bp [5,7]. Studies in the mouse have found similar results, with ~7% of genes possessing upstream antisense transcripts, with most antisense TSS located between 0.1 and 1.0 kb upstream of the coding gene TSS [8]. Interestingly, genes with upstream antisense transcription had a higher level of transcription; however, they also showed an accumulation of immature transcripts, suggesting competition for transcript elongation and splicing factors. Divergent transcription was also shown to be associated with genes involved in transcriptional regulation, in agreement with the observed enrichment of divergent transcription in human transcription factors reported by Li et al. [7]. This is consistent with the proposed role of divergent transcription in enhancing the stochasticity of expression that is often observed in lineage-determining transcription factors [9,10].

## 2. Structural Features of Bidirectional Promoters

**Figure 2:**
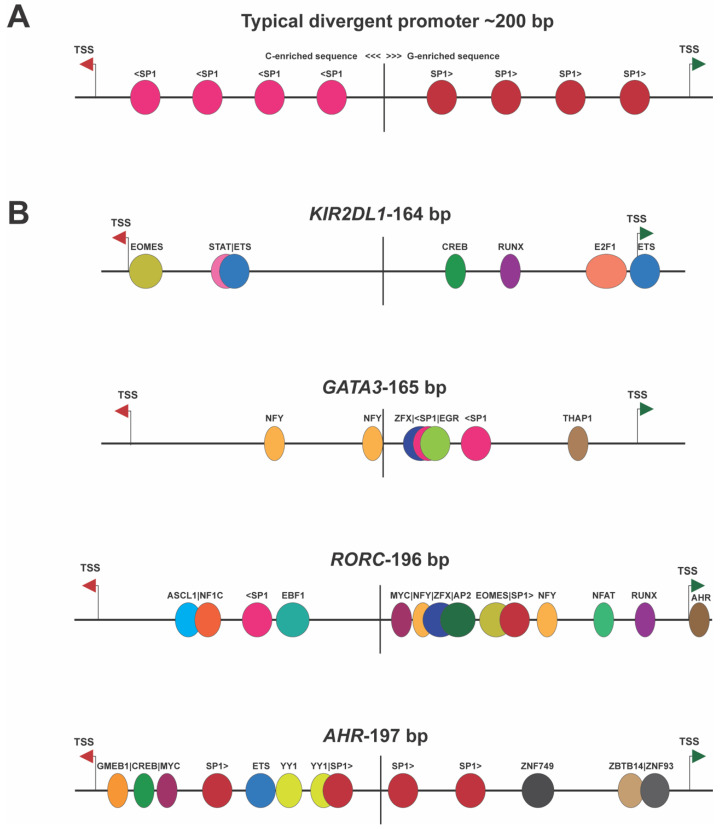
Divergent promoters exhibiting switch behavior lack the symmetry seen in housekeeping genes’ bidirectional promoters. (**A**) Illustration of the symmetrical nature of housekeeping gene bidirectional promoters [1]. The vertical line indicates the middle of the bidirectional element. The antisense TSS is indicated by the red arrow, and a green arrow shows the position of the sense TSS. The left side of the element is C-rich, possessing SP1 sites (light pink) on the antisense strand, and the right side is G-rich, with SP1 sites (red) on the sense strand. (**B**) Examples of bidirectional promoters shown to behave as switch elements. The non-symmetrical arrangement of TF-binding sites is shown for four human genes, with the gene name and separation of TSS shown above each cartoon.

The complete sequencing of the human genome revealed an unexpected abundance of gene pairs arranged in a head-to-head orientation with a shared promoter region [5]. It was previously thought that the close arrangement of genes in operons was limited to organisms with compact genomes, such as prokaryotes and viruses. Divergent gene pairs were frequently observed in human genes involved in DNA repair or other housekeeping functions, with ~23% of the housekeeping genes analyzed having divergent TSS within 300 bp [11]. These bidirectional promoters were found to be G/C-rich, as expected for housekeeping promoters, but they also contained CpG islands that overlapped with the first exons of the two genes. A study of bidirectional promoters in human and mouse genes determined that bidirectional promoters were characterized by variable transcriptional start sites and a defined midpoint at which sequence composition changed strands and divided the element into two halves with opposite directions of transcriptional initiation [1]. There is a group of TF-binding motifs that are enriched in bidirectional promoters: Nuclear Transcription Factor Y (NF-Y), Early 2 Factor 1 (E2F1), Early 2 Factor 4 (E2F4), GA Binding Protein Transcription Factor Subunit Alpha (GABPA), Myelocytomatosis oncogene (MYC), Nuclear Respiratory Factor 1 (NRF1), Specificity Protein 1 (SP1), THAP Domain Containing 11 (THAP11), and Yin-Yang 1 (YY1) [12]. The binding of the ubiquitously expressed Erythroblastosis proto-oncogene (ETS) family member GABP was highly associated with bidirectional promoters, and the insertion of a strict GABP-binding site (CCGGAAGTG) into unidirectional promoters was able to confer bidirectional activity [13]. Bidirectional promoters are generally more active than unidirectional promoters and thus provide an efficient mechanism for the expression of housekeeping genes [11]. However, bidirectional promoters with TSS within 200 bp have also been shown to behave as switch elements, wherein the element alternates between transcription in the sense or antisense direction [7,14,15]. Figure 2 shows the structures of several of these switch elements, demonstrating that they do not possess the symmetrical arrangement of TFs observed in housekeeping genes’ bidirectional promoters. Convergent promoter pairs, wherein the antisense promoter TSS is downstream of the TSS of the gene, will also create a genetic switch since simultaneous bidirectional transcription cannot occur due to the collision of RNA polymerase II complexes. Direct RNA polymerase II competition for binding to DNA would also occur when the TSS are closer than 60 bp since each polymerase molecule covers at least 30 bp upstream of the TSS [16,17]. Therefore, divergent bidirectional promoter pairs with TSS less than 60 bp apart should be functionally considered as belonging to the convergent class of bidirectional promoters with direct physical competition of RNA polymerases (Figure 1C). In contrast to the dynamic competition that would occur in adjacent divergent promoter pairs, convergent transcriptional competition would be expected to result in more stable transcriptional states due to the disruption of competing downstream promoter complex formation by an actively transcribing convergent promoter.

## 3. Variegation of MHC Class I Receptor Expression by Bidirectional Promoters

Although transcription factors constitute a large percentage of the genes containing divergent and convergent antisense, there are clearly many other gene types utilizing this type of regulation. The mouse and human families of major histocompatibility complex (MHC) class I receptors provide a good example of variegated gene expression programmed by competing sense/antisense promoters. The mouse lymphocyte antigen 49 (*Ly49*) gene family is composed of a large number of C-type lectin-related activating and inhibitory receptor genes, and gene content varies dramatically between mouse strains [18]. Individual inhibitory receptors are expressed at differing frequencies on subsets of natural killer (NK) cells in a probabilistic manner, and ~ 80% of mature NK cells express between one and three different Ly49 proteins. The size and composition of the Ly49-expressing NK subsets is consistent within a given mouse strain, indicating genetic programming. The mechanism of this programming has been linked to a bidirectional promoter located ~ 5 kb upstream of the TSS region used to express Ly49 protein in mature NK cells [15]. In contrast to the G/C-richness of most bidirectional promoters, the *Ly49* elements are small 100 bp TATA-based promoters with competing TF-binding sites determining the probability of sense versus antisense transcription. The relative strength of the sense versus antisense promoter elements correlates with the percentage of NK cells expressing a given receptor, indicating that programmed probabilistic promoter competition determines the NK cell fate. Examination of the independently evolved, Ig superfamily-related human Killer cell Immunoglobulin-like Receptor (KIR) family of MHC receptors revealed the presence of antisense promoters in a configuration like that observed in human transcription factors [19]. The proximal *KIR* promoter is bidirectional, and a spliced convergent antisense transcript is generated by a promoter located in the second intron of the *KIR* genes [20]. A direct role of antisense in gene silencing was revealed by the discovery of a 28 bp piwi-interacting RNA (piRNA) from the antisense strand of the proximal promoter region [21]. *KIR* antisense containing the piRNA was capable of silencing KIR expression, and the deletion of the 28 bp element abrogated silencing. The relative activity of sense versus antisense activity of the proximal promoter correlated with the frequency of KIR expression by mature human NK cells, indicating that promoter competition programmed the variegated expression of KIR as seen for the *Ly49* genes.

## 4. GATA Switches and the Role of Noncoding RNA in Differentiation

A model of transcriptional regulation involving the presence of both divergent and convergent antisense transcription near the TSS of a lineage-determining transcription factor has been proposed [7]. Divergent transcription would increase the stochastic generation of variegated TF expression, resulting in multipotent progenitor cells with differing levels of competing TFs. The probabilistic competition of convergent transcription would allow selective silencing of TFs, leading to the exit from the multipotent state, resulting in the differentiation of cells that had dominant expression of a competing lineage-determining TF. The competition between GATA-binding proteins (GATA) GATA1 and GATA2 for erythroblast versus mast cell differentiation is given as an example, where single cells expressing *GATA1* convergent antisense had higher *GATA2* expression and a mast cell phenotype, whereas cells with *GATA2* antisense had higher *GATA1* transcription and an erythroblast phenotype. Interestingly, the convergent *GATA1* antisense transcript splices into the antisense strand of the second and third exons of the upstream Suppressor of Variegation 3-9 Homolog 1 (*SUV39H1)* gene, encoding a histone methyltransferase that trimethylates lysine 9 of histone H3 (H3K9Me3), which leads to gene silencing. The *GATA1* antisense transcript was shown to inhibit SUV39H1 expression in the human K-562 cell line. Additionally, the inhibition of SUV39H1 expression facilitates the generation of induced pluripotent stem cells, and increased H3K9Me3 reduces the plasticity of progenitor cells [22,23]. Therefore, it was proposed that *GATA1* antisense expression may regulate differentiation through the inhibition of both *GATA1* sense and *SUV39H1* transcription [7]. A common regulatory motif for lineage-defining transcription factors was proposed, with a divergent TSS within 70–200 bp upstream from the TF TSS and a convergent TSS within a 2 kb region downstream from the TF TSS (Figure 1E). The relative strength of competing sense and antisense promoters could therefore program the frequency at which specific differentiation events occur. A recent comprehensive evaluation of antisense transcription across multiple species revealed a similar pattern of convergent and divergent antisense TSS near the start site of genes in xenopus, chicken, mouse, and human, with a clustering of divergent antisense within 200 bp and convergent antisense within 2 kb [24].

## 5. Functional Activities of Noncoding Antisense RNAs

There is accumulating evidence that noncoding RNAs represent functional RNAs that act as protein scaffolds and/or nucleic acid-binding regulators. For instance, promoter antisense transcripts can help facilitate changes in the epigenetic landscape of promoter regions and alter gene expression during differentiation, cell migration, and certain disease states [25]. Here, we provide a series of examples that collectively highlight the importance of antisense noncoding RNAs in the regulation of cell fate determination.

### 5.1. Diverse Functions of Antisense Transcripts in the GATA Gene Family

In addition to the switch behavior generated by competing promoters, there are likely functional consequences linked to the expression of the noncoding RNAs generated by these switch elements, as observed with the *GATA1* antisense transcript. GATA3 is a member of the family of zinc-finger transcription factors that bind to the consensus sequence 5′-AGATAA(A/G)-3′. GATA3 is abundantly expressed in various tissues throughout development, including the brain, placenta, and kidney [26]. Its role in T-cell differentiation has been extensively studied, and as the master regulator of Th2 (T helper type 2) lineage commitment, GATA3 suppresses the Th1 pathway while also upregulating the transcription of cytokines IL-4, IL-5, and IL-13 [27,28]. A study by Gibbons et al. [29] revealed that the *GATA3-AS1* transcript participates in chromatin remodeling of the *GATA3* locus via Mixed Lineage Leukemia 1 (MLL1) lysine methyltransferase 2A (KMT2A) recruitment, H3K4 methylation, and R loop formation in the *GATA3-AS1* locus and that this mechanism is necessary for the normal expression of GATA3 and its downstream targets. Without sufficient levels of *GATA3-AS1* transcription and subsequent GATA3 expression, cells are incapable of fully committing to T cell differentiation. Functional roles for antisense transcription have been found for additional GATA family members. A recent study has revealed a role of the *GATA2-AS1* transcript in the hypoxic response, and common single-nucleotide variants in *GATA2-AS1* exons were associated with early-onset coronary artery disease [30]. Another GATA family member implicated in lineage determination is GATA6. The *GATA6* gene is primarily expressed in gonads, heart, and definitive endodermal tissues such as the pancreas [31]. Two recent papers reported that both GATA6 and GATA6-AS1 are upregulated during the differentiation of these tissues and that the knockdown of GATA6-AS1, a long non-coding RNA (lncRNA) sharing a bidirectional promoter region with GATA6, was sufficient to inhibit GATA6 expression and reduce differentiation with no impact on cell pluripotency [32,33]. Jha et al. [32] analyzed cardiomyocyte differentiation and found that GATA6-AS1 knockdown not only reduced GATA6 but also downregulated mesodermal markers, as well as both canonical and noncanonical Wingless-Type MMTV Integration Site (WNT) signaling-related genes and prevented complete cardiomyocyte differentiation. While Yang et al. [33] looked instead at endodermal differentiation and found that the depletion of GATA6-AS1 knocked down GATA6 by decreasing endoderm-specific TFs Sma and Mad-related proteins 2 and 3 (SMAD2/3) ability to bind to and activate sense transcription [33].

### 5.2. SPI1 Antisense Reduces Gene Expression

Spleen Focus Forming Virus (SFFV) Proviral Integration oncogene 1 (SPI1) is a TF essential for normal myelopoiesis and is known to be an important regulator of monocyte differentiation [34]. Two recent studies of SPI1(PU.1) regulation in AML revealed functional roles for a noncoding RNA transcribed from an antisense promoter (asRNA) in the third exon of the gene and a lncRNA termed LOUP that is transcribed from a promoter/enhancer element 17 kb upstream from the SPI1 promoter [35,36]. The asRNA was shown to reduce SPI1 mRNA, and the LOUP lncRNA facilitated the interaction of the upstream enhancer with the SPI1 promoter and stimulated expression. Runt-related transcription factor 1 (RUNX1) regulates the production of these RNAs, and the RUNX1- Eight Twenty One (ETO) fusion protein favored asRNA expression, thus blocking myeloid differentiation. The authors proposed that the competitive interaction of the upstream enhancer with either the proximal or antisense promoter creates a binary on/off switch for either myeloid (SPI1 on) or T cell development (SPI1 off).

### 5.3. Complex Regulation of the Human E-Cadherin Gene

The human E-Cadherin gene (*CDH1*) gene codes for a cell–cell adhesion protein that is essential for epithelial cell differentiation and tumor control. *CDH1* expression is thought to be controlled by two noncoding RNAs (ncRNA) located near the TSS of the CDH1 coding transcript [37,38]. These ncRNAs are also referred to as promoter-associated RNAs (paRNAs). A 456 bp non-polyadenylated antisense RNA is transcribed from the proximal promoter region, initiating at nucleotide −135 relative to the *CDH1* TSS. The core *CDH1* promoter is in a CpG island containing duplicated SP1, NF-Y, and E-Box binding sites. The methylation of the proximal promoter region silences *CDH1* transcription, and the antisense transcript is only detected in cells that possess sense *CDH1* transcripts, indicating that both sense and antisense transcription of the core 135 bp bidirectional promoter are controlled by DNA methylation. A distal sense strand ncRNA originates at nucleotide −720 relative to the *CDH1* TSS. This transcript is also non-polyadenylated, and it terminates just prior to the *CDH1* TSS at positions −54 and −10. In contrast to the strong correlation of antisense transcription from the proximal bidirectional promoter with *CDH1* transcription, the distal ncRNA is also found in cells that do not express *CDH1* mRNA, such as the prostate cancer cell line PC3. An active role of the distal ncRNA in gene silencing was shown by knocking down its expression with siRNA or by interfering with an endogenous silencing mechanism by knocking down the Argonaute RISC Component 1 (*AGO1*) transcript, which is linked with gene silencing through its association with an edited endogenous miRNA, isomiR-4534 that binds to the −118 to −134 region at the 3′ end of the distal sense transcript. This results in the recruitment of SUV39H1, a histone methyltransferase discussed earlier. Furthermore, a single nucleotide polymorphism (SNP) associated with increased susceptibility to prostate cancer, rs16260-A, increases AGO1 binding to the distal transcript, favoring gene silencing. The distal sense transcript was found to be associated with chromatin, consistent with a role in gene silencing. However, the role of the promoter-associated antisense transcript is less clear, with one group finding that the antisense transcript is not chromatin-associated, and its knockdown leads to gene activation, suggesting a role in maintaining gene activity by acting as a “hooking scaffold” that recruits the epigenetic regulators, Ubiquitin like with PHD and Ring Finger domains 1 (UHRF1), DNA Methyltransferase 3 Alpha (DNMT3A), SUV39H1, and Suppressor of Zeste 12 (SUZ12), involved in *CDH1* repression. In contrast, Pisignano et al. [38] reported chromatin association of the antisense transcript. However, neither study addresses the potential role of the physical interaction of the noncoding transcripts and the possibility that they are generating double-stranded RNA. The antisense transcript may prevent the distal transcript from associating with the chromatin, and Magnani et al. [37] show the dissociation of the distal transcript from the chromatin in LNCaP cells that express high levels of antisense transcript. The presence of an antisense transcript may also shield the miRNA binding site on the distal sense transcript, preventing AGO1 binding and gene silencing. A consistent feature of these studies is the inhibitory role of distal sense transcripts and the presence of antisense transcripts when the *CDH1* gene is transcriptionally active. Whether or not the relative levels of proximal promoter sense/antisense transcripts vary during development or under conditions that lead to gene silencing is a matter worthy of further investigation.

### 5.4. Regulation of Gene Expression by lincRNAs

Noncoding RNAs do not need to be directly adjacent to a gene to affect gene expression. Long intergenic noncoding RNAs (lincRNAs) are defined as transcribed RNAs that have no overlap with protein-coding genes and are at least 200 nucleotides in length [39]. LincRNAs differ from smaller RNAs due to their ability to fold and create tertiary structures, giving them a wider range of potential functions. Currently, lincRNAs have been found to be active in a wide variety of processes, such as the maintenance of pluripotency and cell fate commitment programs, and lincRNA dysregulation can result in tumor formation and genetic disorders [40]. Some lincRNAs can directly or indirectly modify gene expression by recruiting chromatin modifying complexes and interacting with transcription factors. Therefore, lincRNAs can have activating or inhibitory roles, and some can even perform both. One of the most well-known lincRNAs, and a good example of the important roles they play in gene regulation, is X-inactive-specific-transcript (*Xist*) RNA, which is responsible for recruiting chromatin-modifying proteins to deactivate one of the two X chromosomes in mammalian females very early in development [40]. To explore the impact lincRNAs can have on maintenance of pluripotency, Guttman et al. [41] performed loss-of-function experiments using short hairpin RNAs (shRNAs) to knock down the expression of dozens of lincRNAs in mouse ES cells and found this was sufficient to decrease expression of pluripotency markers such as the NK2-family homeobox transcription factor Nanog and Octamer-binding transcription factor 4 (Oct4) and upregulate various differentiation programs. The authors proposed that due to the tendency of many lincRNAs to interact with multiple regulatory complexes, they may even serve as cell-type-specific scaffolds. LincRNAs have multiple essential functional roles in gene regulation and are necessary for the regulation of cell fate determination.

## 6. Control of Viral Replication by Bidirectional Promoters

Bidirectional promoter elements can also function as probabilistic switches determining whether a virus actively replicates or downregulates gene expression to avoid detection by antiviral mechanisms of the host. The choice between the lytic and lysogenic fate of bacteriophage lambda provides an early example of this type of programming. Competition between the pL and pR promoters and repressor feedback mechanisms determine the fate of cells infected by the bacteriophage [42]. Similar trends are seen in bacteriophage 186 with the pL and pR transcription start sites overlapping by 62 bp. In bacteriophage 186, pR is able to knock down pL expression 5.6-fold, likely by RNA polymerase elongation over the weaker promoter, while pL was not shown to have any effect on pR expression, highlighting the asymmetric nature of some switches [43]. Antisense transcription is also found to be highly ubiquitous in mammalian viruses such as the Epstein–Barr virus, with as much as 65 percent of its genome transcribed in both directions and many sense and antisense transcripts overlapping [44]. Since the regulation of bacteriophages has been extensively reviewed, we will examine two examples of mammalian viruses controlled by bidirectional promoters.

### 6.1. Polyomavirus

BK polyomavirus (BKPyV) infects a substantial fraction of the human population without producing specific symptoms. However, immunosuppression in kidney transplant patients can permit high-level BKPyV replication, leading to nephropathy or hemorrhagic cystitis [45]. An approximately 400 bp noncoding control region (NCCR) of the viral genome coordinates the temporal regulation of the consecutive steps of early viral gene region (EVGR) expression, viral genome replication, and late viral gene region (LVGR) expression [46]. The large T antigen (LTag) is a major EGVR product that drives genome replication, but it is also a target of the immune system; therefore, its expression must be limited in order to allow viral persistence in immunocompetent individuals. The bidirectional NCCR element controls the relative levels of the divergent EVGR and LVGR transcripts. The NCCR in healthy individuals is associated with slow replication in human renal cells; however, highly replicative variants associated with renal pathology in transplant patients possess nucleotide changes in the NCCR that affect the divergent expression of either the EVGR or the LVGR. There is an imperfect symmetry between the EVGR- and LVGR-proximal parts of the NCCR, consisting of SP1-binding sites, TATA and TATA-like elements, initiator elements, and downstream promoter elements. NCCR mutations that inactivate the SP1 site on the LVGR side of the element lead to greater EVGR expression and increased viral replication. Mutations of the SP1 site adjacent to the EVGR TATA element inactivate the NCCR, inhibiting viral replication. Studies examining the effect of mutating the TATA and TATA-like elements at either end of the NCCR had similar effects. Mutation of the TATA-like element associated with LVGR transcription increased EVGR transcription, and mutation of the EVGR TATA element nearly abolished EVGR expression and reduced LVGR expression to 20% of the normal level. In addition, the bidirectional balance of EVGR and LVGR expression was shown to depend on the affinity, strand orientation, and number of SP1 sites. It therefore appears that the virus makes use of the competition between divergent promoter elements to program the level of replication [47].

### 6.2. mCMV

The murine cytomegalovirus (mCMV) major immediate–early (MIE) enhancer is essential for virus replication by enhancing MIE gene transcription to initiate the viral transcriptional program in acute infection and virus reactivation from latency [48]. The ~700 bp enhancer region is flanked at one end by transcription unit *ie1/3* and at the other end by *ie2*. Transcription occurs in opposite directions, giving rise to spliced transcripts. The IE1 and E3 proteins both act as trans-activators of viral early (E)-phase genes. The IE1 protein is also involved in the early disruption of nuclear domain 10 and the transactivation of cellular genes involved in nucleotide metabolism. The IE2 protein has been shown to be capable of gene transactivation; however, it is dispensable for CMV replication in cell culture. To distinguish between independent or simultaneous activation of *ie1* and *ie2* transcription by the enhancer, a mouse model of CMV latency in the lung was studied, since transcription from the MIE region is a rare event, with only 10 to 20 transcriptional events per 10^6^ latent viral genomes. RT-PCR analysis of lung tissue fragments revealed that *ie1* and *ie2* transcription occurs independently, indicating that the MIE element is functioning as a switch rather than synchronizing the expression of the divergent transcripts [49].

## 7. Conclusions and Future Perspectives

The frequent presence of antisense transcriptional units near the TSS of coding genes indicates a major role of antisense in gene regulation. The use of a single G/C-rich bidirectional promoter element to achieve high-level expression of two linked housekeeping genes is an efficient mechanism that allows comparable expression of genes acting together in a particular pathway. However, the most intriguing aspect of antisense transcription is its potential role in the programming of cell fate. There should be a program encoded in the DNA to produce lineage-determining factors at pre-defined frequencies in order to reproducibly generate specific ratios of different cell types during development. Therefore, the probabilistic competition of pairs of sense and antisense promoters may play a major role in the programming of cell fate. There are many examples of bidirectional promoter elements that can produce differing ratios of sense to antisense transcripts, and these ratios can be programmed by adjusting the relative affinity of competing TF-binding elements that affect the efficiency of sense versus antisense transcription initiation and/or elongation. The observation that lineage-determining TFs are enriched for convergent and divergent antisense transcripts in the TF promoter region provides an opportunity to modulate cell fate determination by mutating key elements involved in the probabilistic choice between sense and antisense transcription. Future work in this area should involve both directed and agnostic approaches to identify promoter switches acting in developmental pathways. 5′-directed single cell RNAseq can be used to discover antisense promoters specifically active in progenitor cell populations, which can then be assessed for their role in the differentiation process. Conversely, progenitor and differentiated cells can be compared to discover changes in antisense transcription that are associated with various differentiated phenotypes. Since there are approximately 300 TFs that contain both convergent and divergent antisense transcripts near the start site of the gene, there are abundant opportunities to discover promoter-driven mechanisms underlying the programming of cell fate.

## Data Availability

Not applicable.
